# A method to establish a c-Myc transgenic mouse model of hepatocellular carcinoma

**DOI:** 10.1016/j.mex.2020.100921

**Published:** 2020-05-17

**Authors:** Yan Mei, Chao Zhou, Chao-Yong Liang, Guan-Ming Lu, Mu-Sheng Zeng, Jin-Jin Wang, Guo-Kai Feng

**Affiliations:** a State Key Laboratory of Oncology in South China, Collaborative Innovation Center for Cancer Medicine, Sun Yat-sen University Cancer Center, 651 Dongfeng East Road, Guangzhou 510060, China; b Department of Medical Oncology, Affiliated Tumor Hospital of Guangxi Medical University, Nanning 530021, Guangxi, China; c Department of Breast and Thyroid Surgery, Affiliated Hospital of Youjiang Medical University for Nationalities, Baise, Guangxi 533000, China; d Shanghai Model Organism Center, Inc, Shanghai, China

**Keywords:** c-Myc, Transgenic mouse model, Hepatocellular carcinoma, Knock-in

## Abstract

Hepatocellular carcinoma (HCC) remains one of the most lethal malignant cancers worldwide. HCC mouse models are widely used to explore the molecular pathogenesis of HCC and to test novel drug candidates.

The advantages of this mouse model are as follows:

• This method developed a H11^LNL-Myc^ knock-in HCC mouse model by crossing H11^LNL-Myc^ heterozygous mice with (albumin (Alb))-cre transgenic mice to generate c-Myc/Alb-cre double positive mice.
• The c-Myc/Alb-cre double-positive mice exhibited a typical HCC phenotype, and showed accelerated tumor initiation and rapid HCC progression. Early stage HCC tumors (2–3 mm in diameter) were observed in male mice at the age of 47 days and in female mice at the age of 60 days.
• Approximately 3 months later, the HCC tumors had progressed to a late stage (> 1 cm in diameter), and 100% of the male and female mice had HCC.

## Graphical abstract

**Figure:**
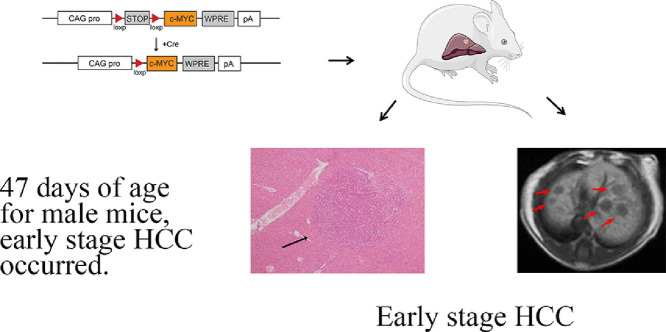

Specifications Table

**Table:** 

Subject area:	Biochemistry, Genetics and Molecular Biology
More specific subject area:	Developing a H11LNL-Myc knock-in HCC mouse model
Method name:	c-Myc knock-in mouse model
Name and reference of original method:	Murakami H., 1993. Transgenic mouse model for synergistic effects of nuclear oncogenes and growth factors in tumorigenesis: interaction of c-myc and transforming growth factor alpha in hepatic oncogenesis. Cancer Res. 53(8):1719–23.
Resource availability:	Shanghai Model Organism Center, Inc,

## Method details

### Introduction

HCC is estimated to be the fifth most common cause of cancer and the second leading cause of cancer-related deaths, and its incidence is increasing worldwide [1]. At the molecular level, previous studies have revealed that c-Myc is frequently overexpressed in HCC. In particular, c-Myc is overexpressed in up to 70% of patients with viral and alcohol-related HCC [2]. c-Myc functions mainly as a transcription factor that coordinates many biological processes, and c-Myc activation contributes to autonomous proliferation and growth [3], inhibition of differentiation [4], and induction of genomic destabilization [5]. c-Myc coordinates the transcription of thousands of protein-coding genes, microRNAs, and long noncoding RNAs [3,^6^,7]. Due to the importance of c-Myc in HCC, c-Myc-targeted therapy should be a promising therapeutic strategy [8] and the c-Myc transgenic mouse model of HCC is an ideal model.

The Cre-loxP system is a powerful technology that is widely used for targeted genome editing. While the Cre-loxP system is predominantly used for targeted gene deletion, it also induces site-specific gene insertion, which is mediated by a Cre-loxP-carrying lentiviral vector [9]. Regarding the mechanism of the Cre-loxP system, a single Cre recombinase recognizes two directly repeated loxP sites, and then the Cre recombinase excises the loxP flanked DNA, thus creating two types of circular DNA, depleted and inactivated targeted genes [10]. Tissue-specific expression of Cre recombinase results in tissue-specific reactivation or inactivation of a target gene. Here, we provide a detailed protocol for the construction of c-Myc transgenic mouse model of HCC and highlight some of its applications.

### Mice and animal care

B6.Cg-Tg(Alb-cre)21Mgn/J mice with a C57BL/6J background were purchased from the Jackson Laboratory (Bar Harbor, ME, USA), and C57BL/6J mice were purchased from the Guangdong Medical Laboratory Animal Center (Foshan, China). The mice were housed under specific pathogen-free conditions in the Animal Center of Guangdong Pharmaceutical University.

### H11^LNL-Myc^ mouse model

**Fig. 1:**
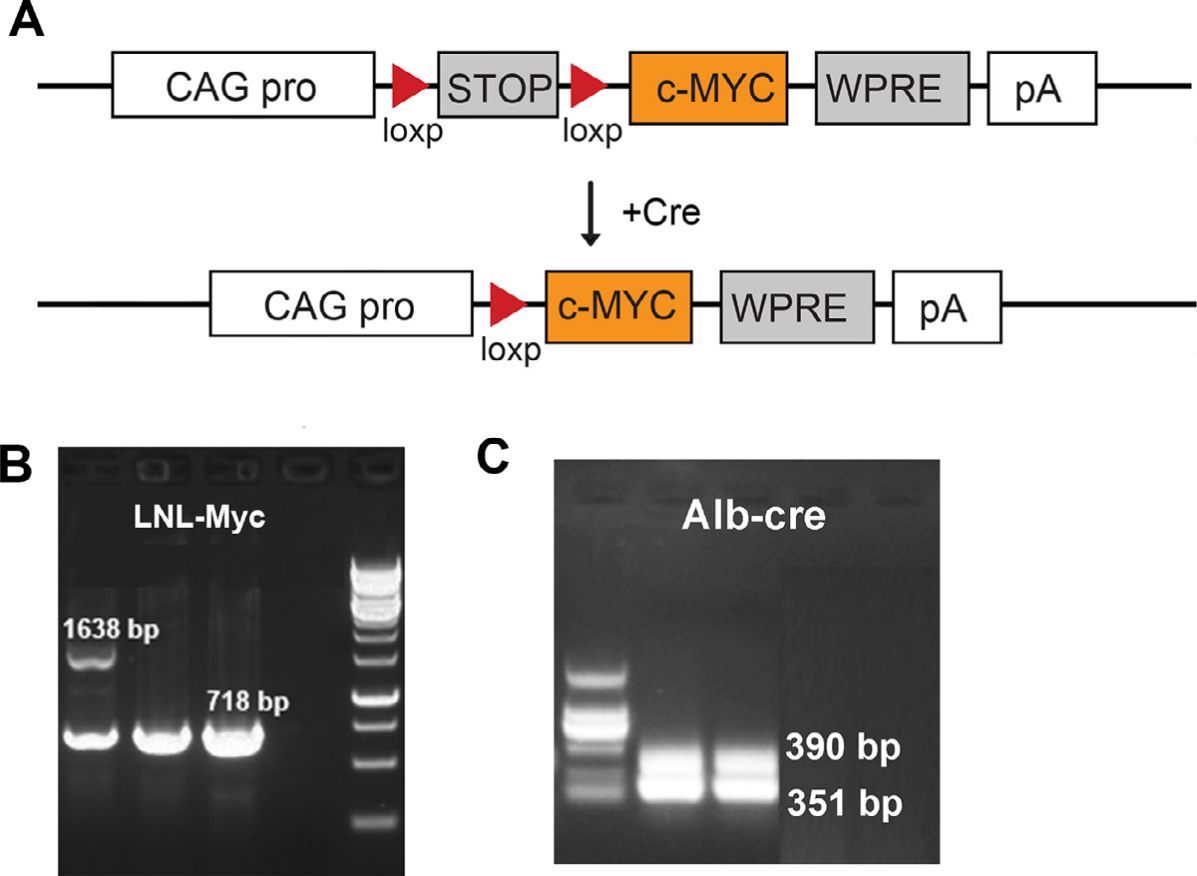
Generation and verification of c-Myc/Alb-cre double-positive mice. (A) The H11^LNL-Myc^ targeting vector was showed at the top, the c-Myc/Alb-cre double -positive mice were generated by crossing H11^LNL-Myc^ heterozygous mice with Alb-cre transgenic mice, and the STOP-loxp fragment was deleted in the c-Myc/Alb-cre double positive mice. (B) PCR amplification of LNL-Myc results in a 1638-bp mutant product and a 718-bp wild-type product. (C) PCR amplification of Alb-cre results in a 390-bp mutant product and a 351-bp wild-type product.

The H11^LNL-Myc^ knock-in mouse model was developed by Shanghai Model Organisms Center, Inc. This model was generated by the CRISPR/Cas9 system on the C57BL/6J mouse background. Briefly, the pCAG-loxp-Neo-loxp-Myc-polyA fragment was inserted into the well-defined *Igs2* locus (the Hipp11 or H11 locus by homologous recombination. The LNL-Myc targeting vector was created with the CMV enhancer/chicken beta-actin core promoter (CAG), a loxp-NEO-loxp cassette (LNL), the Myc gene, the woodchuck hepatitis virus posttranscriptional regulatory element (WPRE) and the SV40 polyA signal (Fig. 1(A)).

Cas9 mRNA was transcribed *in vitro* with the mMESSAGE mMACHINET7 Ultra Kit (Ambion, TX, USA) according to the manufacturer's instructions, and subsequently purified using the MEGAclear™ Kit (ThermoFisher, USA). The sequence of the Cas9 targeted guide RNA (sgRNA) was 5′- ATGATGGCATCTAATGAGCT −3′; this sequence was transcribed *in vitro* using the MEGAshortscript Kit (ThermoFisher, USA) and subsequently purified using the MEGAclear™ Kit. The donor vector carrying sgRNA and Cas9 mRNA was microinjected into fertilized C57BL/6J eggs. The F0 generation mice that were positive for the homologous recombination were identified by long Polymerase Chain Reaction (PCR). The primers (P1–P4) used for genotyping to identify the correct homologous recombination were P1: CTTGTGAGGGCCTACTGTGAC and P2: CTTTCCGGAGATAGGGTGTTA for the correct recombination of the 5′ homology arm, and P3: 5′- TGCCCCTTTGTGTTCTCTTGTAG-3′ and P4: 5′- ATCGTGGGCATGTGACCTCTC-3′ for the correct recombination of the 3′ homology arm. The PCR products were further confirmed by sequencing. The F0 mice were crossed with C57BL/6J mice to obtain H11^LNL-Myc^ heterozygous mice. c-Myc/Alb-cre double-positive mice were generated by crossing H11^LNL-Myc^ heterozygous mice with Alb-cre transgenic mice. In this model, Cre recombinase was expressed under the control of the mouse albumin promoter to achieve hepatocyte­specific overexpression of c-Myc; activated c-Myc signaling was sufficient to induce murine HCC.

### PCR genotyping of the Alb-Cre and c-Myc mice

The Alb-Cre and c-Myc transgenes were detected by PCR genotyping, and the following primer pair was used to genotype the Alb-cre mice: wild type forward: TGCAAACATCACATGCACAC; mutant forward: GAAGCAGAAGCTTAGGAAG-ATGG; and common reverse: TTGGCCCCTTACCATAACTG. These primers amplify a 390-bp fragment in the mutant mice and a 351-bp fragment in the wild-type mice. The following primer pair was used to genotype the c-Myc mice: wild type forward: GGAGGAGGACAAACTGGTCA; mutant forward: TGTCCATTCAAGC-AGACGAG; and common reverse: GGAGGAGGACAAACTGGTCA, these primers were designed to produce a 1638-bp and a 718-bp fragment in the mutant and wild-type mice, respectively (Fig. 1(B)).

### Histopathology

The liver tissues were collected when the female HCC mice reached the ages of 60 and 90 days, which correspond to early stage HCC and late stage HCC, respectively. The tissues were embedded in paraffin, and 3-µm sections were cut, and stained with hematoxylin and eosin (H&E).

### Contrast enhanced magnetic resonance imaging

Mouse Magnetic Resonance (MR) Imaging was performed with a 1 T MR system (Aspect Magnet Technologies Ltd, Netanya, Israel), which was mounted with a 30-mm solenoid Tx/Tr coil (inner diameter 30 mm) and fast gradient coils. The entire abdomen was imaged from the diaphragmatic dome to the inferior margin of the pubic symphysis. Axial T1-weighted spin-echo sequence (TR/TE, 330/10) were obtained before and after tail vein injection of 0.2 mL/kg Gd-EOB-DTPA (Bayer Schering Pharma, Berlin, Germany). Images were obtained using a field of view (FOV) of 30 × 30 mm, matrix of 120 × 120 and slice thickness of 2 mm.

### Statistical analysis

The survival analyses were carried out using GraphPad Prism (version 7.0), and Kaplan–Meier curves and log-rank tests were used for the survival analyses.

### Early stage HCC in the c-Myc/Alb-cre transgenic mice

**Fig. 2:**
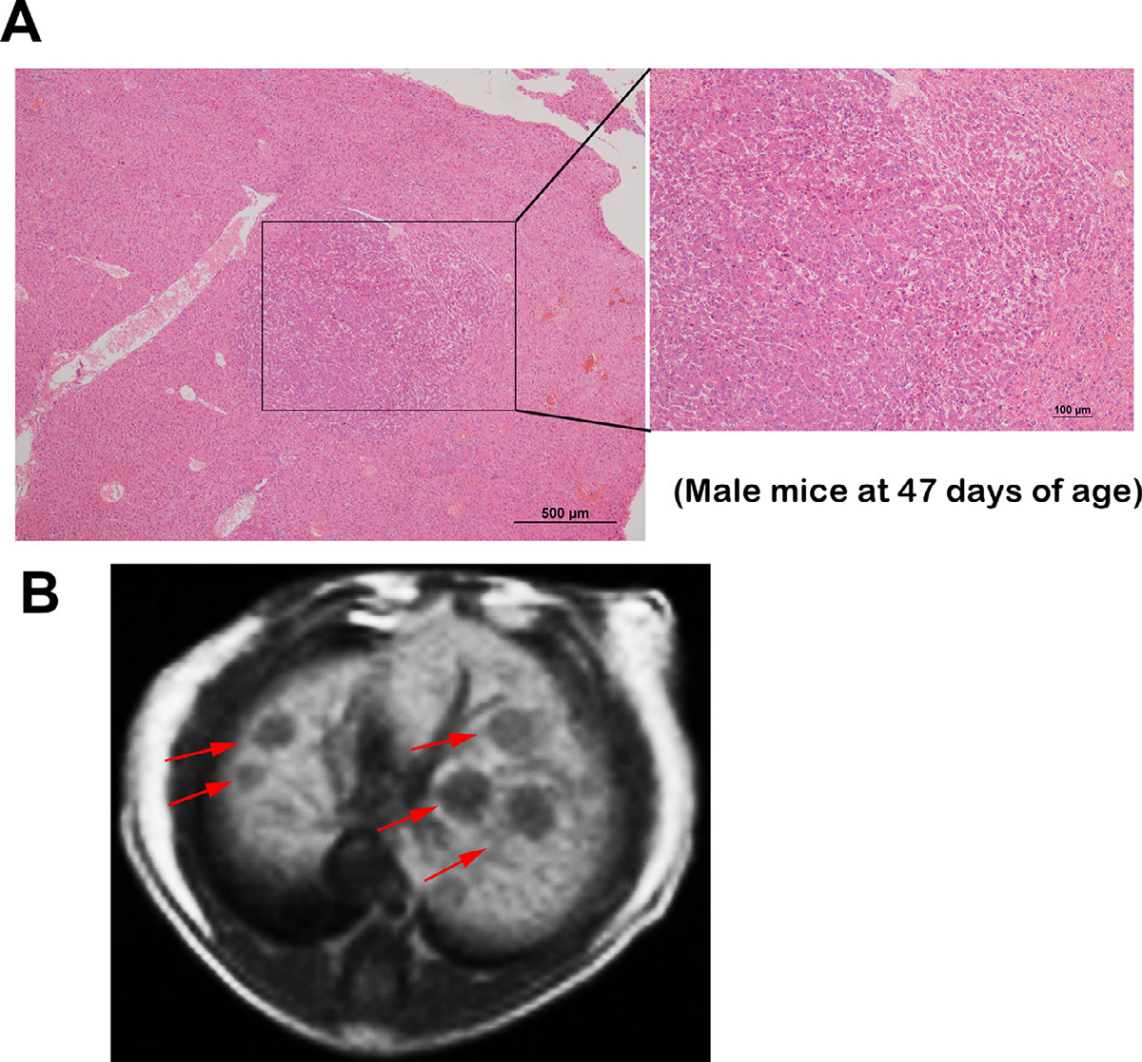
Early stage HCC in c-Myc/Alb-cre transgenic mice. (A) Representative images of the HE-stained early stage HCC tumors from a c-Myc/Alb-cre transgenic male mouse at the age of 47 days. (B) Representative MR imaging of the early stage HCC tumors from a c-Myc/Alb-cre transgenic male mouse at the age of 47 days with the hepatobiliary MR contrast agent gadoxetate disodium Gd-EOB-DTPA. The HCC lesions are marked by red arrows.

The c-Myc signaling pathway was hepatocyte­specific activated, and HCC progressed rapidly and with a high frequency in the c-Myc/Alb-cre transgenic mice. Early stage HCC tumors (2–3 mm in diameter) were observed at the age of 47 days for male mice and 60 days for female mice (Fig. 2(A)); 83.33% (5/6) of the male mice and 66.67% (4/6) of the female mice had early stage HCC as determined by MR imaging with the hepatobiliary MR contrast agent gadoxetate disodium Gd-EOB-DTPA (Fig. 2(B)).

### Late stage HCC in the c-Myc/Alb-cre transgenic mice

**Fig. 3:**
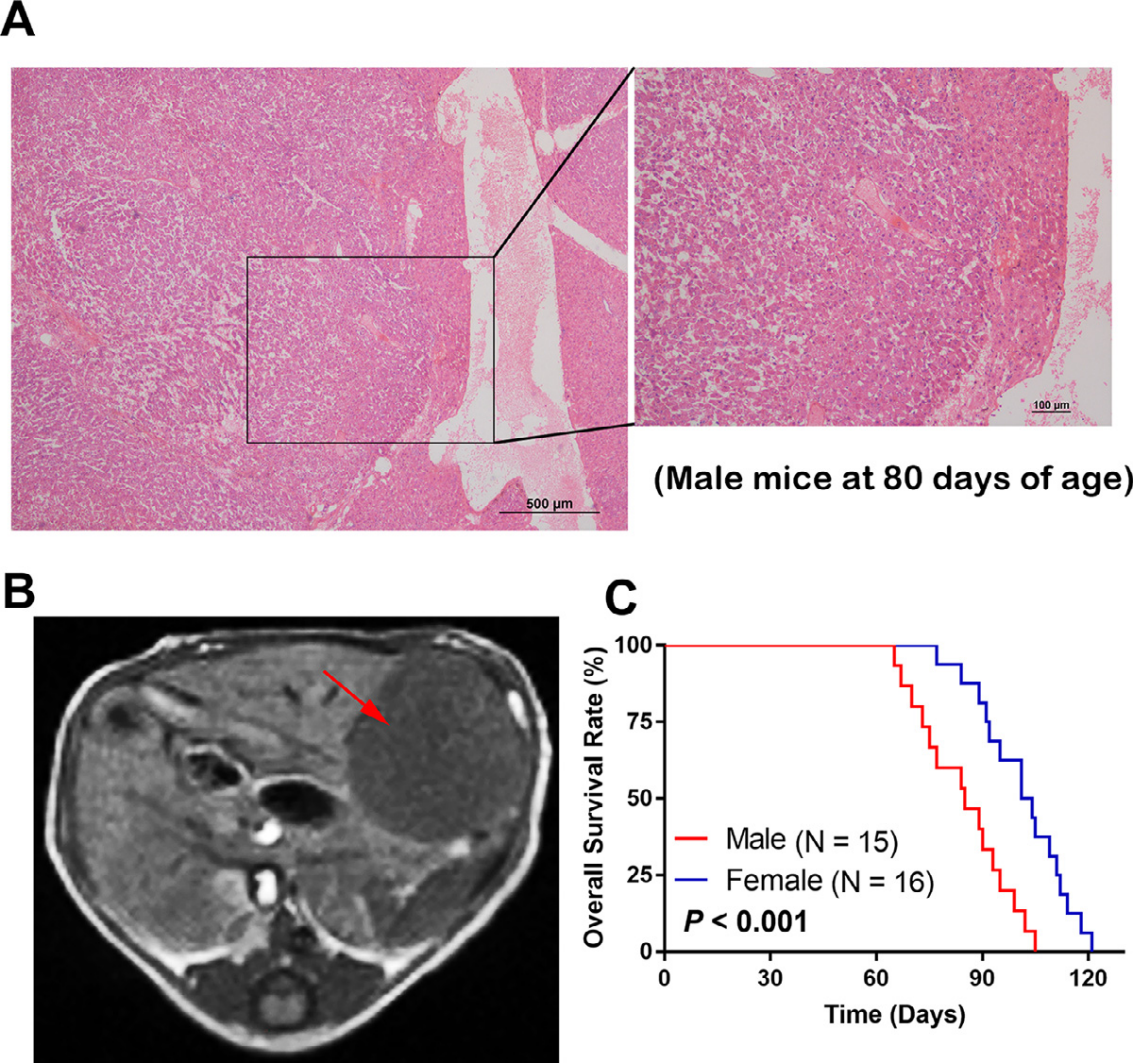
Late stage HCC in c-Myc/Alb-cre transgenic mice. (A) Representative images of the HE-stained late stage HCC tumors from a c-Myc/Alb-cre transgenic male mouse at the age of 80 days. (B) Representative MR imaging of the late stage HCC tumors from a c-Myc/Alb-cre transgenic male mouse at the age of 80 days with Gd-EOB-DTPA. The HCC lesion was marked by a red arrow. (C) The Kaplan–Meier curves showing the overall survival of the female (*n* = 16) and male (*n* = 15) mice.

After approximately three months, HCC tumors had progressed to a late stage (> 1 cm in diameter), and 100% (7/7) of the male and (6/6) of the female mice had HCC (Fig. 3(A and B)). The survival times were significantly different between the male and female transgenic mice, and the mean survival times were 85 days for the male mice and 102.5 days for the female mice (Fig. 3(C)).

## Conclusions

In this study, we established the H11^LNL-Myc^ knock-in HCC mouse model. The c-Myc/Alb-cre double positive mice showed accelerated tumor initiation and rapid HCC progression. A total of 83.33% (5/6) of the male mice and 66.67% (4/6) of the female mice had early stage HCC within 2 months after birth, and 100% of the mice had late stage HCC within 3 months after birth. Murakami et al. generated double transgenic mice overexpressing c-Myc and TGF-α in the liver, and found that 100% of the male and 30% of the female Albc-Myc/MT-TGF-α mice developed HCC within 8 months after birth [11,12]. Another commonly used mouse model is the diethylnitosamine (DEN) induced HCC mouse model, in which DEN is typically administered to male mice at 15 days of age by a single intraperitoneal injection (25 mg/kg). Abnormal foci, nodules and adenomas are detected by ~6 months, and HCC tumors appear at ~10 months [13].

HE staining and MR imaging showed that our mouse model (c-Myc/Alb-cre) exhibited a typical HCC phenotype, and the male mice showed earlier tumor initiation and HCC progression than the female mice. In humans, the incidence of HCC in men is more than twice that in women [14], and in other experimental models of hepatocarcinogenesis, HCC develpos developed more frequently in male mice than in female mice. In a mouse model of DEN induced HCC, Naugler et al. showed that the tumor appears in 100% of the males but only in 13% of the females [15].

In conclusion, our mouse model (c-Myc/Alb-cre) exhibits a typical HCC phenotype, and it exhibits earlier tumor initiation and faster HCC progression compared with other HCC mouse models. This mouse model is an ideal model for studying of the mechanisms of c-Myc driven hepatocarcinogenesis and c-Myc-targeted therapy.

## Declaration of Competing Interest

The authors confirm that there are no conflicts of interest.
